# Occult orbital penetrating injury by plastic straw hidden in birthday cake: A case report

**DOI:** 10.1016/j.ijscr.2025.111797

**Published:** 2025-08-12

**Authors:** Shelly Rauser, Dalbert Chen, Douglas Van Putten

**Affiliations:** a Loma Linda School of Medicine, 11175 Campus St, Loma Linda, CA 92350, USA; b Loma Linda Eye Institute, 11370 Anderson St, Suite #1800, Loma Linda, CA 92354, USA

**Keywords:** Foreign body, Occult, Orbital injury, Penetrating injury, Plastic straw, Oculoplastic orbital surgery

## Abstract

**Introduction:**

Occult foreign bodies consist of foreign material that remains undetected in the body for weeks, months, or even years. In this case report, we document a unique case in which a patient retained a hidden, occult orbital plastic foreign body for eighteen months following a trauma during which the patient's face struck into a birthday cake.

**Presentation of case:**

A 33-year-old male presented to the emergency department with acute right periorbital swelling, pain, and erythema eighteen months after his face struck into a birthday cake. Eighteen months prior, the patient reported experiencing only a small superonasal eyelid laceration at the time of the trauma with no other symptoms and stated that the laceration healed nicely following the incident. Upon examination in the emergency department, a blue plastic straw was observed protruding superonasally from the right upper eyelid, accompanied by periorbital edema, erythema, and purulent fluid emanating from the straw. The size and location of the straw was confirmed with computerized tomography of the orbit. The patient underwent surgical removal of the 28 mm foreign body and recovered well.

**Discussion:**

Orbital foreign bodies can present asymptomatically, with sometimes only a minor laceration as a clue. If the foreign body misses crucial structures of the orbit, the patient will retain full extraocular motion and vision until the body begins rejecting the material. The body may not begin to reject the material until weeks, months, or years later if the foreign body is of an inert material such as plastic. This case underscores the importance of obtaining a thorough history and maintaining a high index of suspicion for foreign bodies of the orbit after a trauma.

**Conclusion:**

This case provides insight into the dangers of smashing faces into cakes as a prank or as a celebration, as there are often support structures in the cake that pose dangers to the anatomy of the face, including the globe and orbit. This case report adds to the minimal amount of existing literature regarding plastic orbital foreign bodies.

## Highlights

• Occult foreign bodies are defined as asymptomatic and undetected by the patient.
• The superonasal region of the orbit asymptomatically tolerates foreign bodies well.
• Foreign bodies of an inert material can go undetected for weeks, months, or years.
• Support structures in cakes pose a severe threat to the globe and orbit.
• Smashing faces into cakes as a prank or a celebration can result in severe trauma.

## 1 Introduction

Foreign bodies can oftentimes go undetected if the material is nonorganic, such as glass, plastic, or metal, and if the object does not cause damage to important surrounding structures [1]. If an inert foreign body is asymptomatic aside from a laceration at the point of entry, it can go undetected for weeks, months, or even years [1]. These hidden, asymptomatic objects are known as occult foreign bodies [2]. In this case, we present a patient who had an occult plastic straw that remained undetected for eighteen months after the original trauma. This case report has been reported in line with the SCARE checklist [3].

## 2 Presentation of case

A 33-year-old male presented to the emergency department with acute right periorbital swelling, pain, and erythema. He reported a history of trauma eighteen months earlier, during which his face struck into a birthday cake. At that time, a small laceration of the right upper eyelid was noted, and the patient presented to an outside emergency department. Per the patient's report, no sutures were required for the laceration, no imaging was performed, and he was subsequently sent home with reassurance. The laceration healed without any apparent complications. Sixteen months after the initial trauma, the patient noticed a blue plastic straw protruding from his right upper eyelid at the site of the previous laceration. The patient was unable to remove the straw himself despite multiple attempts and instead began trimming the straw as it extruded over time. The straw remained in the same position for an additional two months, but the patient did not seek medical attention until the recent worsening of swelling and pain, which prompted the patient to present to the emergency department.

**Fig. 1:**
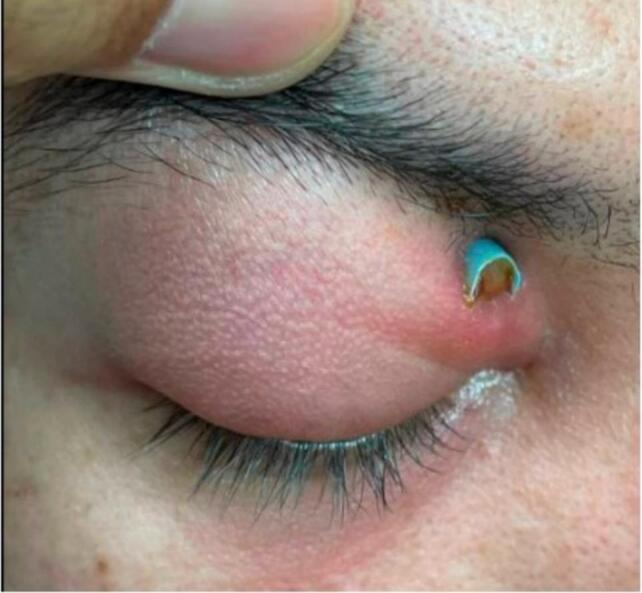
Image taken upon presentation to emergency department. Blue plastic straw visualized protruding out of the orbit. (For interpretation of the references to colour in this figure legend, the reader is referred to the web version of this article.)

**Fig. 2:**
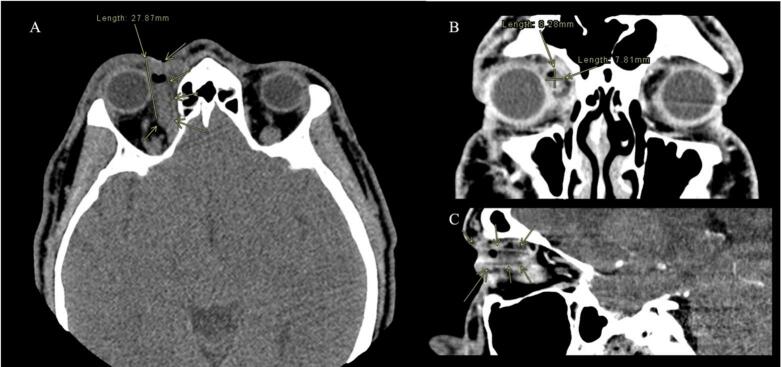
CT of the orbit demonstrating the penetrating straw. A) Axial view of orbit. Arrows pointing to tubular foreign body with a length of 27.87 mm. B) Coronal view of orbit. Arrows pointing to tubular foreign body with two diameter measurements of 8.28 mm and 7.81 mm. C) Sagittal view of orbit. Arrows pointing to tubular foreign body.

On initial examination, best corrected visual acuity (BCVA) was 20/20, intraocular pressure was normal, and the pupils were equal, round, and reactive to light bilaterally. On the external exam, the confrontation visual field test was normal bilaterally, and a blue plastic straw was observed protruding superonasally from the right upper eyelid, accompanied by periorbital edema, erythema, and purulent fluid emanating from the straw (Fig. 1). The patient denied blurry vision, diplopia, or pain with eye movement. The right eye revealed −1 superior duction but was otherwise full in extraocular movements. The rest of the physical exam showed normal anterior and posterior eye findings with an intact globe. Computerized tomography (CT) of the orbit showed a tubular appearing density within the right medial extraconal tissues, superomedial to the medial rectus muscle and medial to the superior oblique muscle. It measured approximately 28 mm in length and 8x8mm axially with associated findings of right extraconal orbital cellulitis (Fig. 2). The patient was started on a two-week course of oral amoxicillin-clavulanate and metronidazole antibiotics.

**Fig. 3:**
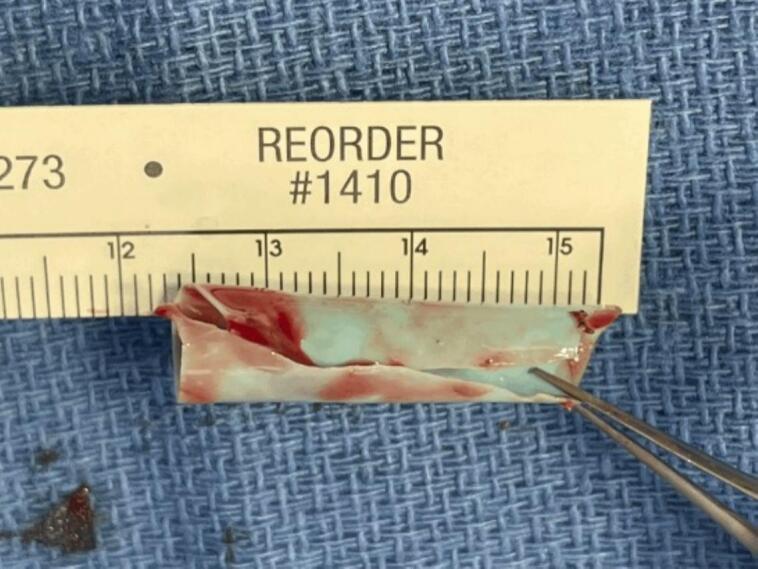
Straw that was removed during surgery measuring about 30 mm. Longitudinal split visualized down the middle.

**Fig. 4:**
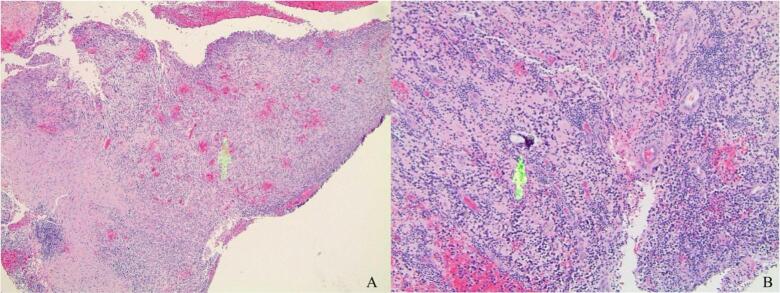
Intra-straw tissue biopsies. A) Arrow pointing to granulation tissue. B) Non-granulomatous and granulomatous inflammation with arrow pointing to a piece of possible foreign body.

**Fig. 5:**
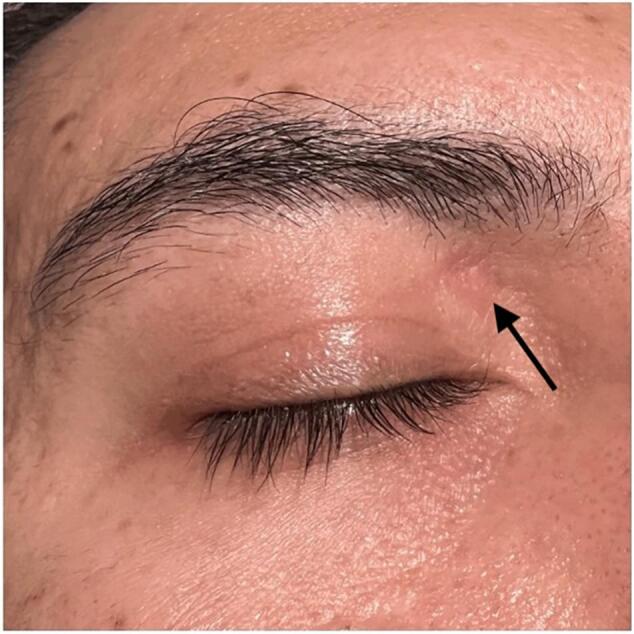
Final healed image; Arrow pointing to surgical scar.

Two weeks later, the patient underwent surgical removal of the foreign body. The plastic straw, found to be split longitudinally, was successfully extracted and measured to be 30 mm (Fig. 3). During the procedure, the tissue within the straw was carefully dissected out and sent for histopathological analysis, revealing both non-granulomatous and granulomatous inflammation (Fig. 4). At the patient's twelve-week post-op visit, he demonstrated complete healing with a BCVA of 20/20 and full extraocular motion bilaterally (Fig. 5).

## 3 Discussion

Penetrating orbital foreign bodies can sometimes go undetected when fully embedded in soft tissue, especially if they avoid critical surrounding structures. These types of orbital foreign bodies are known as occult injuries when the original laceration does not initially draw suspicion for a foreign body, often leading to the discovery of the foreign body later in the future due to complications [2]. In this case, the only notable clue to the foreign body was a bleeding laceration at the time of the incident, which is characteristic of superior orbital foreign bodies [4]. The superonasal region is especially well tolerated if the object misses surrounding structures, exemplified by the plastic straw narrowly missing the superior oblique and the medial rectus muscles, preventing significant extraocular motion deficits or diplopia, which might have otherwise pointed to a retained foreign object. As a result, the laceration healed over the plastic straw, and the straw went undetected for 18 months. This case underscores the importance of obtaining a thorough history and maintaining a high index of suspicion for orbital foreign bodies, as they can present asymptomatically.

In the aftermath of a retained foreign object, the object may be discovered through the formation of a granuloma as the body starts to reject the material. The body mounts an inflammatory response against foreign objects as it attempts to break down and digest the material, creating layers of granulomatous tissue around the object, examples of which were seen in the histopathological samples from this case. Organic foreign bodies, such as wooden foreign bodies, may elicit a faster immune response from the body than inorganic foreign bodies such as glass, plastic, or metal, which are usually more inert [1]. For example, one published case reported a glass foreign body of the eyelid retained for 13 years [1]. In our case, the inert nature of the plastic straw likely contributed to its prolonged retention without detection. Additional complications of foreign body retention include infection in the following weeks or months of implantation. The patient in this case noted the plastic straw gradually protruding through his eyelid, and the resulting infection ultimately brought him into the emergency department to seek medical care.

This case provides additional insight into the dangers of smashing faces into cakes as a prank or as a celebration. Cakes made by professional bakers often have support rods or straws inserted into them to add support and structure to the cake. Such objects pose a significant threat to the ocular and orbital region, potentially resulting in ruptured globes or permanent vision loss. One published case documented a 14-year old girl who sustained an orbital injury after her face was smashed into a tiered cake embedded with wooden dowels [5]. Our case highlights that plastic straws in addition to wooden dowels are being used as support structures in cakes, and consumers should exercise caution. To our knowledge, this is the first published report detailing the consequences of plastic straws embedded in cakes. Fortunately, the patient in this case experienced full recovery, retaining a complete range of extraocular motion and excellent visual acuity.

## 4 Conclusion

Orbital foreign bodies can present asymptomatically with only a minor laceration as a clue. Therefore, it is important to obtain a thorough history and maintain a high index of suspicion for foreign bodies of the orbit after a trauma. If the foreign body was missed when the trauma first occurred, it can be retained in the body for many months, as it was in this case. This case offers both public health precautions regarding smashing faces into cakes as well as adds to the minimal amount of existing literature regarding plastic orbital foreign bodies.

## Ethical approval

This study is exempt from ethical approval at our institution because it is a case report.

## Patient consent

Written informed consent was obtained from the patient for publication of this case report and accompanying images. A copy of the written consent is available for review by the Editor-in-Chief of this journal on request.

## Funding

This research did not receive any specific grant from funding agencies in the public, commercial, or not-for-profit sectors.

## Author contribution

Shelly Rauser: Data collection, writing the paper

Dalbert Chen: Study concept and design, data collection, writing the paper

Douglas Van Putten: Study concept and design

## Guarantor

Shelly Rauser

## Research registration number

Not applicable

## Conflict of interest statement

This research did not receive any specific grant from funding agencies in the public, commercial, or not-for-profit sectors. The authors report no conflicts of interest. All authors attest that they meet the current ICMJE criteria for authorship.
